# Occult olfactory neuroblastoma presenting with multiple bone metastases: a case report

**DOI:** 10.1097/MD.0000000000022630

**Published:** 2020-11-25

**Authors:** Qi Zhou, Zijian Li, Bei Liu, Long Zhao, Baohong Tian, Lina Wang, Yaming Xi

**Affiliations:** a The First Clinical Medical College, Lanzhou University; b Department of Hematology, The First Hospital of Lanzhou University; c Department of Oncology, Donggang Branch of The First Hospital of Lanzhou University.

**Keywords:** Bone marrow metastasis, occult olfactory neuroblastoma, positron emission computed tomography scan

## Abstract

**Rationable:**

Olfactory neuroblastoma (ONB) is a rare malignant tumor of the nasal cavity, the primary local symptoms are usually inconspicuous. Patients are often admitted to various specialties based on different primary symptoms, which may result in delayed diagnosis and even a misdiagnosis.

**Patient concerns:**

Here we report a case of ONB that presented initially as multiple ostealgia without any local symptoms of the tumor and primarily misdiagnosed as multiple myeloma. The patient was a 47-year-old female with bone pain at multiple sites. The initial diagnosis was considered as multiple myeloma. However, the morphologic examination of bone marrow suggested that the tumor cells originated from the nervous tissues. After the positron emission computed tomography scan, the primary lesion in the nasal cavity was located, and a biopsy was performed.

**Diagnosis:**

The final diagnosis of ONB was confirmed by histopathological tests.

**Interventions:**

The patient was treated with metronomic chemotherapy.

**Outcomes:**

The symptoms of bone pain were significantly relieved 3 months later. The emission computed tomography scan of the whole body bones and the magnetic resonance imaging of the head showed that the tumor size did not change significantly and proved a progression-free of the disease.

**Lessons:**

It is a reasonable strategy to identify the original latent tumor by a prompt positron emission computed tomography scan when the primary diagnosis indicates a metastatic disease, especially for the occult malignancies like ONB.

## 1 Introduction

Olfactory neuroblastoma (ONB) accounts for approximately 3 to 5 percent of all cases of malignancies in the nasal cavity.^[1]^ There is no difference in the incidence between genders or among races.^[2]^ ONB can occur at any age; the highest incidence is in the sixth decade.^[3]^ Due to the multidirectional differentiation characteristics, the exact origin of ONB cells is yet unclarified. The classification of neck tumors of 2005 defined that ONB cells originate from olfactory epithelial basal cells.^[4]^ In the early stage of the disease, ONB mostly confined to the nasal cavity and usually showed no significant symptoms or just some non-specific symptoms, such as epistaxis, nasal obstruction, olfactory decline or loss, watery eyes, and blurred vision.

Due to the latent course of ONB, metastases of this disease are common at diagnosis. The most common metastatic sites are neck lymph nodes and bones with vertebral bone as the most common victims.^[5]^ Metastasis to the liver, lungs, brain or breast had also been reported as an anecdote.^[6]^ The extensive infiltration of bone marrow by ONB cells can cause manifestations as anemia, thrombocytopenia, leukocytopenia, or pancytopenia that usually indicates hematologic malignancies and cause misdiagnosis for the absence of symptoms or signs of the primary disease. Positron emission computed tomography (PET-CT) can identify tumor lesions that can not be detected by a computer tomography (CT) scan, including either local recurrence or distant metastasis.^[7]^ The histopathologic test is a recognized gold standard for the diagnosis of a tumor, including ONB.

Because of the rare incidence and the early distant metastasis, the primary local symptoms of ONB are usually inconspicuous. Therefore, ONB patients are often admitted to various specialties based on different symptoms of the metastasis site, which may result in delayed diagnosis and even a misdiagnosis.^[8]^ Thus, it is crucial to keep vigilance and take a promptly complete assessment for patients with malignancies such as ONB. Considering the uncertainties of diagnostic direction, an initial PET-CT scan is a relatively effective and sensitive screen strategy for occult malignancies.

## 2 Case report

A 47-year-old female was transferred to our hospital for lumbosacral bone pain for 2 months that gradually aggravated to chest and backbone. She was diagnosed as multiple myeloma (MM) by a local hospital for the growing bone pain and extensive lesions of the 3^rd^, 4^th^ and 5^th^ lumbers, bilateral femoral bones, and pubic bones, which indicated by an magnetic resonance imaging (MRI) scan. The physical examination revealed pallid lips and conjunctiva, a hard, smooth, painless and a mildly enlarged spleen about 3 centimeters under subcostal and tenderness of the spine, bilateral ribs, and sternum.

The complete blood cell counts panel of the patient showed pancytopenia. The red blood cell counts at the level of 3.08 × 10^12^/L with Hemoglobin at 89 g/L. The white blood cell counts showed at a level of 7.18 × 10^9^/L with a differential count of neutrophil 57.8%, lymphocyte 30.1%, monocyte 10.7%. The platelet number at a level of 54 × 10^9^/L.

**Figure 1:**
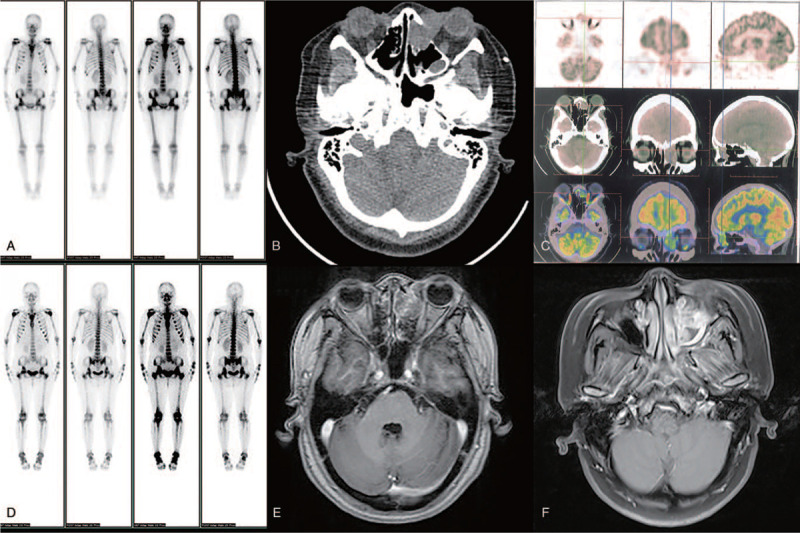
The ECT scan showed that the abnormal distribution of many lesions in the bilateral humerus, sternum, ribs, thoracic vertebrae, lumbar vertebrae, pelvis, and bilateral femurs (A). PET-CT scan showed changes in the left nasal cavity of the patient (B). PET-CT showed increased shadow metabolism of soft tissue density. Its intake of tracer 18F-fluorine deoxyglucose was 6.8 units (C). The ECT crown position of the whole body bone after chemotherapy (D). After chemotherapy, the skull MRI examination axis T1WI showed a slightly lower signal for the tumor. The signal is lower than the brain (E). The MRI of the skull after chemotherapy showed that the axis T2WI showed a tumor signal higher than the brain parenchyma (F).

The biochemistry test proved an increased serum lactate dehydrogenase and β2-microglobulin at the level of 492.0 U/L and 4797.0 ng/ml. Quantity of serum immunoglobulin and complement were in normal range. The serum protein electrophoresis and immunofixation electrophoresis were normal. The whole body bone emission computed tomography (ECT)showed activated metabolism in the bilateral humerus, sternum, ribs, thoracic vertebrae, lumbar vertebrae, pelvis and bilateral femur proximal bone, which indicated MM might be (Fig. 1A).

**Figure 2:**
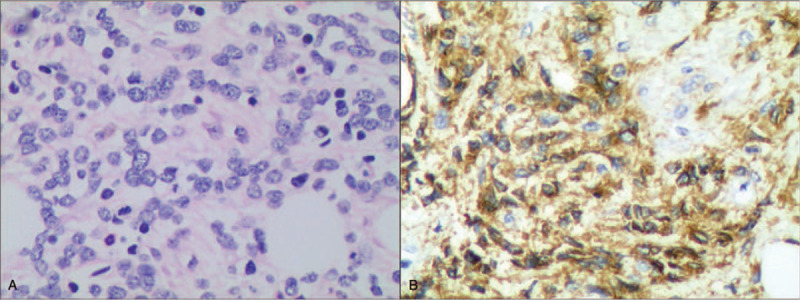
Myelopsy Tissue Pathology shows infantile cell proliferation, distribution in pieces, local focal tissue hyperplasia (HE staining × 400) (A). CD56 expressed in the cell membrane and cytoplasm of the bone marrow tumor cell (immunohistochemical staining × 400) (B).

However, examination of bone marrow did not support the diagnosis of MM. The smear of bone marrow aspiration discovered a small number of unidentified cells in a hypoplasia background without any abnormal cells with charicteristics of MM. Bone marrow biopsy showed a hypoplasia marrow with regional proliferation of immature cells and fibrosis (Fig. 2A). The immunohistochemistry test of bone marrow revealed that the diffused neoplastic cells are positive for CD56, CD34, CD117, CD3, CD20, CD79a, CD68 and negative for CD45, CD138, cytokeratin pan, epithelial membrane antigen, chromophilic A, Synaptophysin (Syn), indicated a nerve originated metastatic tumor (Fig. 2B).

In order to identify the primary disease, PET-CT scan was carried out in the patient. It was found the soft tissue in the left nasal cavity and maxilla with bone destruction (Fig. 1B and C), mild splenomegaly with slightly enhanced uptake.

**Figure 3:**
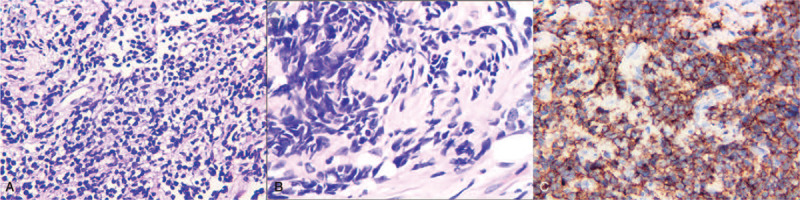
Pathological biopsy of the left nasal pharyngeal wall tumor shows that most tumor cells had the same size and shape, and the cell volume was small. The background of the grid-like nerve fibers was visible between the tumor cells (HE staining × 400) (A). Some tumor cells are larger in size and arranged in a solid nested cable. Epidermal characteristics (HE staining × 400) (B). Cell membrane and cytoplasm diffuse positive of CD56-labeled tumor cells (immunohistochemical staining × 400) (C).

Based on the findings of the PET-CT scan, fiberoptic pharyngorhinoscopy found a polypoid, grayish-red and fragile tumor with a smooth surface and bleeding tendency on the left posterior pharyngeal wall. Biopsy of this tumor showed flaky or striated cancer cells distributed among fibers and collagen with similarly small size and a rounded or irregular shape, abundant or few cytoplasm with distributed vacuoles under the cell membrane with prominences, hyperchromatic nuclear and differently dyed nucleolus. The grid-like nerve fibers distributed among the tumor cells (Fig. 3A and B). The immunohistochemistry tests proved that the cancer cells is positive for CD56 (+) (Fig. 3C), S-100 protein (S-100) (partial +), Ki 67 (+85%), CD57 (+), and negative for glial fibrillary acidic protein, CgA, P40, human melanoma black, CD20, CD3, Syn, T-cell intracellular antigen-1, Growth factor receptor-binding protein, CD43, CD30, cytokeratin pan, neuron-specific enolase and CD138. This pannels is consistent with olfactory neuroblastoma. Therefore, the patient was diagnosed as olfactory neuroblastoma (Kadish stage: D stage) and chemotherapy was initiated immediately.

The patients received chemotherapy (intravenous cyclophosphamide 0.4 g on the 1st and 8th day, and intravenous pirarubicin 50 mg on the 8th day). The symptoms of bone pain were relieved without any special discomfort. At the end of the third chemotherapy cycle, the whole body bone ECT scan (Fig. 1D) and the head MRI (Fig. 1E and F) were reexamined. Compared with the pre-treatment imaging data, there was no significant change in tumor size, indicating no disease progression. The patient is still in follow-up.

## 3 Discussion

The ONB is occult and usually grows slowly in the nasal cavity but tends to invade the adjacent tissues and transfer distantly in the very early stage without local symptoms.^[9]^ The distant metastasis usually occurs in lymph nodes, bloodstream, or cerebrospinal fluid.^[10]^ Vertebral metastasis is the most common bone metastasis.^[11]^ The patient we reported here presented as extensive progressive bone pain and pancytopenia, not the space-occupying symptoms of primary tumors such as epistaxis, nasal obstruction, or hyposmia. Both the symptoms and the extensive bone marrow lesions suggested by the initial MRI and ECT scan indicated a diagnosis of hematologic malignancy like MM. Fortunately, the bone marrow examination indicated that the cancer cells were non-hematologic originated and the diagnostic strategy was redefined to screen the primary malignancy. PET-CT scan identified the primary lesions promptly to obtain the pathological evidence that confirmed the diagnosis of ONB.^[11]^ Therefore, PET-CT screening of primary tumors is crucial for the correct diagnosis of the patient.

PET-CT is widely used in the evaluation of tumors such as staging and monitoring of relapse and metastasis, shows higher sensitivity than the traditional imaging examination. Here, we give the patient a PET-CT examination, identified the tumor in the nasal cavity and the bone leisons caused by it. ONB is a occult cancer with highly malignant and invasive, so early detection and therapy are of great importance. From this example, it can be seen that PET-CT can provide important diagnostic clues for olfactory neuroblastoma to make early diagnosis.^[12]^

Kadish firstly proposed the principle of staging of ONB.^[13]^ In the Phase A, the tumor is restricted to the nasal cavity. In the stage B, the tumor invade in 1 or more nasal sinus. In the stage C, the tumor infiltrates the adjacent tissues or metastasize to the distant tissues. Morita proposed the “improved Kadish” staging by adding stage D that includes all disease with distant metastasis.^[14]^ Stage C was redefined to the disease with continuous infiltration and without any skip. This case belongs to the improved Kadish stage D. Because of the heterogeneity, the improved Kadish staging showed no value to guide the choice of treatment for ONB. Although it is easy to miss a diagnosis, for the identified tumor, conventional imaging tests such as CT and MRI can clearly show the tumor boundary, brain parenchyma invasion and the fine structure within the tumor.

The pathologic examination is decisive to diagnose ONB. The typical ONB tumor cells are similar small round cells.^[15]^ The poorly differentiated ONB cells usually form well-defined nestlike masses among matrix composed by neurofibrils can be described as Flexner-Wintersteiner chrysanthemum masses or Horner-Wright pseudo-chrysanthemum masses, the well-differentiated ONB lacks these traits and immunohistochemical identification is required.^[16]^ The neurogenic molecular markers that can distinguish ONB from hematologic malignancies include CD56, S-100, neuron-specific enolase, Syn, chromophilic A and neurofilament.^[17,18]^ As mentioned above, the patient was positive for CD56 and S-100 and was diagnosed as ONB.

There is no standard treatment for ONB for its rarity and lacking proofs supported by randomized controlled clinical trials. For disease in a very early stage, resection of the tumor is optimal. Post-operative adjuvant radiotherapy can reduce the local recurrence. In cases of regional invasion and distant metastasis, chemotherapy can improve the quality of life and extend survival time.^[19]^ In this case we reported here, extensive bone infiltration made us chose the cyclophosphamide and pirarubicin (CA) regime for chemotherapy. After continuous chemotherapy, the complete blood cell counts gradually recovered and the tumor growth was inhibited, which indicated that CA regime is valid to treat ONB.

Hyams proposed a Hyams staging system^[20]^ containing tumor biological factors and histopathological staging in 1988. The Hyams staging system classifies the disease to low-level (I and II) and high-level tumors(III and IV) according to the biological factors such as lobular structure, Flexner-Wintersteiner or Horner-Wright chrysanthemum clusters, mitosis, nuclear polymorphism, primary fiber matrix, necrosis and calcification. This classification based on the identifiable and reliable degree of differentiation and has become an important basis for treatment choice and provides reliable information for prognosis.^[21]^

The prognostic factors of this disease include age, Hyams histopathological grading, cervical lymph node metastasis, treatment, and proliferation index.^[22,23]^ Ki-67 > 10% usually suggests a poor prognosis.^[24]^ In our patient, bone and bone marrow metastasis, Hyams Histopathology classification IV, Ki-67 over 85% and increased serum lactate dehydrogenase suggested a poor prognosis. Through effective treatment, the 5-year survival rate of patients with olfactory neuroblastoma can reach 70%.^[25]^ Follow-up is required throughout the patient's life.^[26]^ It is recommended that patient should be followed every 4 months in the first 2 years after diagnosis, every 6 months for the next 3 years, and then every 9 months.^[27]^

## 4 Conclusions

Because of the scarce incidence and the inconspicuous local primary manifestations, the probability of misdiagnosis and missed diagnosis of olfactory neuroblastoma is high. As bone marrow are infiltrated, the clinical manifestations are similar to hematologic tumors. Fortunately, the typical morphologic phenotype and molecular markers of ONB cells can distinguish it from a hematologic tumor. PET-CT scan can be used to search lesions that are easy to be missed by traditional imaging technology, and guide a prompt biopsy to achieve early detection, diagnosis and treatment for improvement of the prognosis of the disease.

## Author contributions

**Conceptualization:** Bei Liu.

**Data curation:** Long Zhao.

**Formal analysis:** Zijian Li.

**Investigation:** Long Zhao.

**Methodology:** Zijian Li, Yaming Xi.

**Resources:** Baohong Tian.

**Supervision:** Zijian Li, Yaming Xi

**Validation:** Qi Zhou, Yaming Xi.

**Visualization:** Lina Wang.

**Writing – original draft:** Qi Zhou.

**Writing – review & editing:** Qi Zhou.
